# Sublethal Effects of Imidacloprid on the Population Development of Western Flower Thrips *Frankliniella occidentalis* (Thysanoptera: Thripidae)

**DOI:** 10.3390/insects10010003

**Published:** 2019-01-01

**Authors:** Yu Cao, Hong Yang, Jun Li, Chun Wang, Can Li, Yulin Gao

**Affiliations:** 1 Guizhou Provincial Key Laboratory for Rare Animal and Economic Insect of the Mountainous Region, Department of Biology and Engineering of Environment, Guiyang University, Guiyang 550005, China; yucaosuccess@126.com (Y.C.); Yang18798686746@163.com (H.Y.); C12138259@163.com (J.L.); trade0219@sina.com (C.W.); 2 State Key Laboratory for Biology of Plant Diseases and Insect Pests, Institute of Plant Protection, Chinese Academy of Agricultural Sciences, Beijing 100193, China

**Keywords:** thrips pest, neonicotinoid, phenotypic characterization, sublethal concentration, population fitness

## Abstract

The Western flower thrips (WFT, *Frankliniella occidentalis*) is a global polyphagous pest that is often dependent on chemical control. Imidacloprid has been a commonly used chemical insecticide for effective control of WFT. Low concentrations of insecticides can have sublethal effects on individual insects. However, no more information is known about the effects of exposure at low concentrations of imidacloprid on WFT. Here, we evaluated the effects of imidacloprid at sublethal concentrations on WFT population growth parameters. We first exposed the parental generation to LC_10_ (56.8 mg/L) and LC_25_ (79.2 mg/L) concentrations of imidacloprid. We then quantified various parameters related to the development, survival, and fecundity of the F_1_ generation also exposed to these same concentrations. The development time of the treatment groups exposed to imidacloprid was significantly shorter than the control group, and the net reproductive rate (*R*_0_) was significantly higher for treatment groups than for the control group. For both variables, there was no significant difference between LC_10_ and LC_25_ exposure. The generational survival rate was significantly higher for the control group, followed by the LC_10_ treatment group and then the LC_25_ treatment group. However, the opposite was true for fecundity and intrinsic rate of increase (*r_m_*) of WFT. In summary, exposure to low concentrations of imidacloprid was positive for the population development of WFT, which may contribute to the development of insecticide resistance and cause resurgence in WFT populations.

## 1. Introduction

The Western flower thrips (WFT), *Frankliniella occidentalis* (Pergande) (Thysanoptera: Thripidae), is one of the most destructive and economically important pests of vegetables, fruit, and ornamental crops [1]. In addition to causing direct damage by feeding, WFT cause detrimental indirect damage to crops by transmitting plant viruses, including the tomato spotted wilt virus and impatiens necrotic spot virus [2,3]. WFT is native to Western North America. However, they have attained a worldwide distribution in the past 30 years, and been reported as an invasive pest [1,4].

In China, the WFT was first reported in Yunnan Province in 2000 and then in Beijing in 2003 [5]. It has spread rapidly to most areas of China and has caused severe economic damage [4,6]. Presently, chemical insecticides are the most effective means to control WFT. However, the abuse or intensive use of insecticides causes insecticide resistance in WFT populations and, as a result, control measures for this pest are becoming less effective [7].

Low concentrations of insecticides can have sublethal effects on insects and are often involved in the development of insecticide resistance [8,9,10]. Sublethal effects of insecticides can inhibit or stimulate the growth of insect populations via their effects on development, survival, and reproduction rate of insects, which vary according to the species and the insecticide used [11,12,13]. Thus, it is necessary to study the sublethal effects of insecticides on target insect populations in order to better understand resistance development as well as to devise appropriate strategies for the sustainable control of pests. Despite its pest status and resistance development, the sublethal effects of insecticides on WFT populations have been rarely reported [14].

Imidacloprid was developed in the 1990s and used worldwide to control insect pests that damage host plants by sap-sucking [15,16]. Apart from directly inducing mortality, sublethal effects of imidacloprid have been reported on several insect pests such as *Sogatella furcifera* and *Nilaparvata lugens* [17,18]. Specifically, sublethal concentrations can affect important life history parameters of insect pests, for example by decreasing the fecundity of the F_1_ generation [18,19,20].

Imidacloprid, a nicotine-based insecticide, is one of the most successfully commercialized insecticides against many organisms including thrips pests [21]. Additionally, the resistance of WFT to imidacloprid increased slower and decreased faster compared with resistance to other active ingredients like phoxim and emamectin benzoate [22]. Therefore, imidacloprid was proven to be powerful for the management of WFT in China [22,23,24]. Here, we evaluated the effects of exposure to low concentrations (LC_10_ and LC_25_) of imidacloprid on the development, survival, and fecundity of the F_1_ generation of WFT. Our results explore the mechanisms of pest resurgence induced by insecticides, and also provide important information on the scientific application and administration of imidacloprid for WFT control.

## 2. Materials and Methods

### 2.1. Insects, Plants, and Insecticides

WFT populations were originally collected from various flowering plants in the Guiyang area of Guizhou Province, China in 2014, and were used to establish a laboratory colony [25]. The insects were subsequently maintained on flowers of *Rosa cvs* (Rosales: Rosaceae) free of any insecticides under a 16L:8D photoperiod at 26 ± 1 °C and 60 ± 5% RH.

*Rosa cvs* were grown in greenhouses in the nursery of the Department of Biology and Engineering of Environment, Guiyang University, Guizhou Province, China. The greenhouses were maintained free from insect pests by covering the vent openings with insect-proof netting, and plants were cultivated without the application of pesticides [26]. Flowers at anthesis with intact petals were collected from the *R. cvs* plants for the laboratory experiments.

70% Hezhan WG (the active ingredient is imidacloprid with 70% content; WG = Water disperse granule) was purchased from Shanghai Heben Pharmaceutical Ltd., Shanghai, China.

### 2.2. Sublethal Effects of Imidacloprid on WFT

LC_10_ and LC_25_ concentrations of imidacloprid were previously calculated to be 56.8 and 79.2 mg/L, respectively [24]. Exposure of WFT adults or larvae to LC_10_ and LC_25_ concentrations of imidacloprid was performed using the same leaf dipping method as described previously [24]. To assess the effects of exposing WFT to low concentrations of imidacloprid, we monitored several population growth parameters commonly used for thrips: Development, survival, and fecundity of the F_1_ generation.

We prepared three separate plastic insect-proof containers (20 cm × 14 cm × 9 cm, two experimental containers, and one control container) with *R. cvs* flowers in each container for thrips rearing of the parent generation [25]. Two experimental containers (LC_10_ container and LC_25_ container) received treatment with imidacloprid at LC_10_ and LC_25_ concentrations by leaf dipping method [24], respectively, while the third container contained untreated flowers and served as the control. Intact flower petals were prepared and dipped for 10 s in the LC_10_ and LC_25_ concentrations of imidacloprid with the adaxial surface facing down. Then, the flower petals were air dried for about 30 s and used for thrips rearing in these containers. The same was done to the flowers of the control group, which were dipped in distilled water. Approximately 100 WFT adults (males and female) that were previously maintained on imidacloprid-treated (LC_10_ or LC_25_) flowers according to the method of Cao et al., were introduced into each experimental container [24], with LC_10_ adults introduced into the LC_10_ container and LC_25_ adults into the LC_25_ container. The same numbers of untreated WFT adults were introduced into the control container.

Adults were allowed to mate and oviposit in these three containers. After 12 h, the WFT adults were removed. Because eggs are laid inside the flower tissue and are not visible, the egg’s developmental period was determined by recording the passage of time from removal of adults until the appearance of larvae. As soon as the eggs hatched in the original containers, the newly emerged larvae were placed on fresh flower discs to observe the development periods of each stage of WFT [26]. One hundred flower discs each containing a single flower petal were prepared for each treatment and control group, respectively, with one larva placed on each flower disc, and all discs kept separately under a 16L:8D photoperiod at 26 ± 1 °C and 60 ± 5% RH (relative humidity). Then the developmental stage, from the first instar to adult, was observed daily, and juvenile survival was assessed every 12 h on flower discs under the microscope. The flower discs were prepared by using *R. cvs* petals (~2 cm diameter), which were dipped for 10 s in the insecticide solution (at LC_10_ and LC_25_ concentrations of imidacloprid, respectively) or in distilled water as a control and air dried [24]. Test flower discs (experimental groups and control group) were replaced with fresh discs daily, each with three replicates for a total of 300 larvae for each group.

Newly emerged adult WFT of the F1 generation (from the two experimental groups and control group, respectively) were collected and paired in a glass cylinder (40 mm diameter × 50 mm height) containing one flower petal (with LC_10_, LC_25_ concentrations of imidacloprid treated for experimental groups and untreated for the control group, respectively) for oviposition. The flower petals were changed daily, and the replaced petals were individually transferred to Petri dishes (40 mm diameter) for egg-hatching [26,27]. The Petri dishes were examined to determine the fecundity of WFT, calculated as the number of eggs that hatched daily throughout the lifetime of each female. The offspring were reared to adults for sex determination, and the numbers of female and male offspring were recorded to estimate their sex ratio. Reproduction assays were performed on three replicates of 20 male-female pairs per treatment (a total of 60 pairs per treatment or 180 pairs in total). A life table was constructed according to the method of Nielsen et al. [28] and Cao et al. [26], incorporating all of these related parameters according to the observations.

### 2.3. Statistical Analysis

Data were analyzed using SPSS software (version 18.0; SPSS, Chicago, IL, USA). One-way ANOVA followed by Tukey’s HSD for multiple comparisons were used to compare development time, survival rate, fecundity, sex ratios, and life table parameters of WFT among the various treatments (control, imidacloprid LC_10_ and LC_25_). Life table parameters, including the net reproductive rate (*R*_0_), intrinsic rate of increase (*r_m_*), finite rate of increase (λ), generation time (*T*), and doubling time (DT) were calculated according to Nielsen et al. [28] and Cao et al. [26].

## 3. Results

### 3.1. Sublethal Effects of Imidacloprid on WFT Development

Exposure of WFT to the low concentrations of imidacloprid shortened the duration of the first instar, second instar, and prepupal stages when compared with the control group (*F* = 10.607, *df* = 2732, *p* = 0.011; *F* = 21.053, *df* = 2669, *p* = 0.002; *F* = 7.938, *df* = 2645, *p* = 0.021, respectively) (Table 1). However, developmental time from egg to the first instar and pupal to adult stages did not differ significantly among treatments. There was no significant difference in developmental time from egg to adult between the two treatment groups exposed to imidacloprid (LC_10_ = 9.57 d and LC_25_ = 9.38 d), but both groups had significantly shorter developmental times from egg to adult than the control treatment group (10.31 d; *F* = 51.814, *df* = 2618, *p* < 0.001).

### 3.2. Sublethal Effects of Imidacloprid on WFT Survival

**Figure 1:**
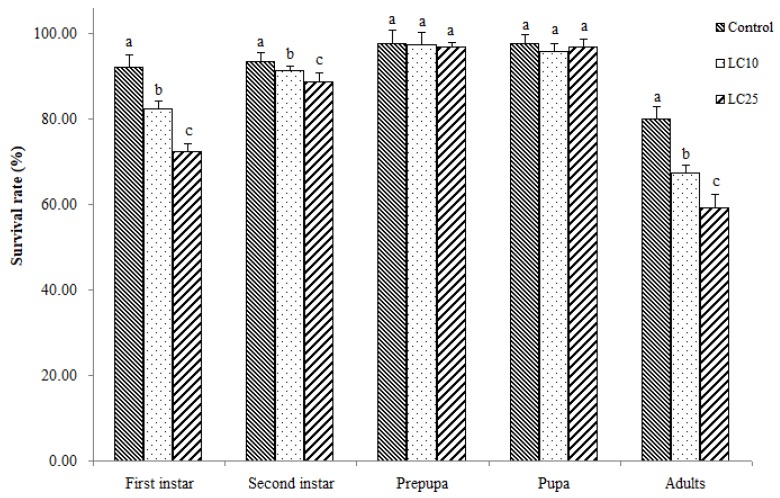
Survival rates (%) of developmental stages of *Frankliniella occidentalis* after exposure to sublethal concentrations of imidacloprid. Data are shown as mean ± SE. Different letters above bars indicate significant differences (one-way ANOVA followed by Tukey’s HSD tests, *p* < 0.05).

The percentage survival of first instar and second instar WFT larvae was significantly reduced following exposure to LC_10_ and LC_25_ imidacloprid concentrations as compared with unexposed WFT, with the control group >LC_10_ exposure and >LC_25_ exposure (*F* = 237.364, *df* = 2732, *p* < 0.001; *F* = 55.380, *df* = 2669, *p* < 0.001, respectively) (Figure 1). However, prepupal and pupal stages did not differ significantly in their survival percentages among exposure and control conditions. Adult WFT survival percentage decreased significantly with increased exposure to imidacloprid in the following order: Control (80% ± 2.33%) >LC_10_ exposure (67.33% ± 2.54%) >LC_25_ exposure (59.33% ± 3.17%) (*F* = 127.478, *df* = 2618, *p* < 0.001).

### 3.3. Sublethal Effects of Imidacloprid on WFT Longevity, Oviposition and Sex Ratios

Female WFT longevity did not differ significantly among the LC_10_ and LC_25_ treatments, nor did it differ among the LC_10_ treatment and control. However, the longevity of female adults in the LC_25_ treatment group was significantly shorter when compared with the control (*F* = 16.923, *df* = 2180, *p* = 0.003) (Table 2). For male WFT, longevity significantly decreased in the LC_10_ and LC_25_ treatment groups as compared with the control (*F* = 17.961, *df* = 2180, *p* = 0.003).

The oviposition period did not differ significantly among the LC_10_ treatment and control groups, both of which were significantly longer than that of the LC_25_ treatment group (*F* = 72.687, *df* = 2180, *p* < 0.001). The highest fecundity was observed in the LC_25_ treatment (82.20 ± 0.64), followed by LC_10_ treatment (79.02 ± 0.44), and then the control group (72.65 ± 0.38) (*F* = 146.977, *df* = 2180, *p* < 0.001). Similarly, both groups of WTF treated with imidacloprid had significantly higher oviposition rates than that of the control group (*F* = 13.286; *df* = 2180; *p* = 0.006).

The sex ratios were 2.07 ± 0.03, 3.34 ± 0.05, and 3.91 ± 0.02 in the control, LC_10_ treatment, and LC_25_ treatment groups, respectively (*F* = 552.166, *df* = 29, *p* < 0.001), with values indicating the ratio of female offsprings to total offsprings.

### 3.4. Sublethal Effects of Imidacloprid on WFT Life Table Parameters

Both groups treated with imidacloprid had significantly higher *R*_0_ than the control group, but there was no significant difference in *R*_0_ between the LC_10_ and LC_25_ treatment (*F* = 15.756, *df* = 29, *p* = 0.004). *R*_0_ was 42.26 ± 0.33 in the LC_10_ treatment, 40.90 ± 0.28 in the LC_25_ treatment, and 38.80 ± 0.25 in the control, respectively (Table 3). Similarly, *r_m_* values increased significantly with increased exposure to imidacloprid, and *r_m_* values were 0.181 ± 0.000, 0.171 ± 0.000 and 0.154 ± 0.000 (*F* = 35.681, *df* = 29, *p* < 0.001) in the LC_25_, LC_10_, and control groups, respectively. The *T* and *DT* of WFT were significantly shorter in both imidacloprid treatment groups compared with the control (*F* = 25.951, *df* = 29, *p* = 0.001; *F* = 21.875, *df* = 29, *p* = 0.002, respectively), and *λ* was significantly higher in the treatments exposed to imidacloprid than in the control (*F* = 15.158, *df* = 29, *p* = 0.005).

## 4. Discussion

Our results revealed that the two low concentrations of imidacloprid (LC_10_ and LC_25_) could give rise to positive effects on the F_1_ generation of WFT, which may have critical implications for the management of WFT. After exposure to low concentrations of imidacloprid at LC_10_ and LC_25_, our results showed that WFT had significantly lower survival rates but significantly faster development and higher fecundity of the F_1_ generation when compared with the control. Significantly faster development periods, higher fecundity, *R*_0_, and *r_m_* were observed when WFT were treated with sublethal concentrations of imidacloprid, which may partially explain the resurgence of thrips after some insecticide use [17,29,30]. Insecticides applied under field conditions may be formulated at effective concentrations, but these concentrations may decrease under field conditions due to natural precipitation, evaporation, or degradation of the insecticides themselves [11]. Thus, insects may effectively be exposed to sublethal concentrations of insecticides in these situations.

In this study, exposure to sublethal concentrations stimulated reproduction and boosted the population growth of WFT. Additionally, sublethal exposure to imidacloprid also led to a higher sex ratio of female offspring, which may also exacerbate population development of WFT. As a typical *r*-strategy pest, the more female WFT present, the faster the reproduction, which is a useful strategy for the survival and maintenance of insect populations in response to insecticide exposure or other adversity. Furthermore, insects could develop resistance following exposure to insecticides at low concentrations [8,9]. Therefore, it is possible that WFT will benefit from the effects of sublethal exposure to insecticides by developing resistance [10,14,31], making effective management of this pest species more difficult.

In contrast, Gong et al., showed that exposure to LC_25_ concentrations of spinosad could significantly inhibit the population growth of WFT [14], which indicates that different types of insecticides may affect the same insect species in different ways. This could also occur when the same insecticide is applied to different insect species. For example, sublethal concentrations of imidacloprid stimulate the population development of *Myzus persicae* but inhibit the population growth of *Nilaparvata lugens*, *Apolygus lucorum* and *Sitobion avenae* [32,33,34,35,36]. Similarly, exposure to low concentrations of cyantraniliprole had a markedly negative impact on *Spodoptera exigua* population growth but a positive influence on *Bactrocera dorsalis* [37,38]. Such negative and positive impacts have been reported from a range of insecticides at sublethal concentrations when applied to diverse insects [39,40]. Thus, the effects of exposure to sublethal concentrations of insecticides on the population development of insects are species- and insecticide-dependent [11].

To further determine the underlying mechanisms of such differences in WFT, more insecticides at low concentrations should be used on this pest to comprehensively understand their effects. Additionally, various concentrations of insecticides should also be used, because different concentrations can have different effects even on the same pest species. For example, *Aphis glycines* had significantly lower fecundity when exposed to 0.20 mg/L imidacloprid via the leaf-dipping method, but higher fecundity when exposed to 0.05 mg/L imidacloprid [41]. Similarly, WFT laid more eggs when exposed to the LC_10_ concentration but fewer eggs when exposed to the LC_25_ concentration of spinosad, as compared with the control [14]. WFT should be exposed to long-term, sublethal concentrations of imidacloprid to comprehensively assess these effects. In this study, we only observed the effects of imidacloprid on the F_1_ generation of WFT. However, Gong et al. reported that negative effects were found in the first generation after LC_25_ treatment of spinosad, but after 32 generations of LC_25_ treatment, they observed increases in growth and reproduction of WFT [14]. A similar phenomenon was also found in *Daphnia carinate* exposed to low concentrations of chlorpyrifos [42].

Low concentrations of insecticides not only affect the biology and physiology of insects but also alter their behavior [11]. For example, a low dose of cyantraniliprole increased the mating competitiveness of treated *B.* *dorsalis* [38]. A low concentration of imidacloprid significantly influenced the probing, settling, and feeding behaviors of WFT, but did not reduce the vector’s transmission of tomato spotted wilt virus [43,44]. However, in *F. fusca*, the low dose of imidacloprid did reduce the transmission of tomato spotted wilt virus in addition to altering its feeding behavior, which was similar to the effect of cyantraniliprole on WFT [44]. Therefore, low concentrations of cyantraniliprole might help to protect agricultural crops and impede disease transfer by controlling WFT, as imidacloprid did for *F. fusca*. The behavioral parameters of these related thrips species resulting from sublethal effects of insecticides also require further investigation.

## 5. Conclusions

Exposure of WFT to low concentrations of imidacloprid at LC_10_ and LC_25_ concentrations led to positive effects for insect development and egg-laying, which significantly stimulated the growth of WFT populations. Imidacloprid induced hormesis effects on the population development of WFT, and significantly higher fecundity, *R*_0_, *r_m_* and other important indexes of population growth were observed in the LC_10_ and LC_25_ imidacloprid treated groups. Therefore, the sublethal effects of imidacloprid should be carefully evaluated when used for WFT control in the field.

## Figures and Tables

**Table 1:** Developmental duration (days; mean ± SE) from egg to adult of *Frankliniella occidentalis* following exposure to sublethal concentrations of imidacloprid.

Stage	Control	LC_10_	LC_25_
Egg	2.59 ± 0.03 a	2.55 ± 0.02 a	2.51 ± 0.03 a
First instar	2.20 ± 0.02 a	1.99 ± 0.02 b	1.96 ± 0.01 b
Second instar	2.88 ± 0.03 a	2.46 ± 0.02 b	2.44 ± 0.02 b
Prepupa	1.32 ± 0.00 a	1.21 ± 0.00 ab	1.15 ± 0.00 b
Pupa	1.33 ± 0.00 a	1.30 ± 0.00 a	1.30 ± 0.00 a
Egg to adult	10.31 ± 0.16 a	9.57 ± 0.14 b	9.38 ± 0.12 b

Different letters in the same row indicate significant differences (one-way ANOVA followed by Tukey’s HSD tests, *p* < 0.05).

**Table 2:** Longevity, fecundity oviposition, and sex ratios of *Frankliniella occidentalis* after exposure to sublethal concentrations of imidacloprid.

Parameters	Control	LC_10_	LC_25_
Longevity/female (day)	26.15 ± 0.27 a	25.20 ± 0.14 ab	24.25 ± 0.51 b
Longevity/male (day)	15.09 ± 0.13 a	13.37 ± 0.83 b	12.68 ± 0.75 b
Oviposition period (day)	22.05 ± 0.48 a	21.15 ± 0.32 a	20.95 ± 0.95 b
Fecundity (first instars/female)	72.65 ± 0.38 c	79.02 ± 0.44 b	82.20 ± 0.64 a
Oviposition rate (first instars/female/day)	3.33 ± 0.04 b	3.78 ± 0.05 a	3.95 ± 0.03 a
Sex ratio of offspring (females/total)	2.07 ± 0.03 c	3.34 ± 0.05 b	3.91 ± 0.02 a

The data are shown as the mean ± SE. Different letters in the same row indicate significant differences (one-way ANOVA followed by Tukey’s HSD tests, *p* < 0.05).

**Table 3:** Life table parameters of *Frankliniella occidentalis* after exposure to sublethal concentrations of imidacloprid.

Parameters	Control	LC_10_	LC_25_
Net reproductive rate (*R*_0_)	38.80 ± 0.25 b	42.26 ± 0.33 a	40.90 ± 0.28 a
Intrinsic rate of increase (*r_m_*)	0.154 ± 0.000 c	0.171 ± 0.000 b	0.181 ± 0.000 a
Mean generation time (*T*)	23.76 ± 0.42 a	21.88 ± 0.36 b	20.50 ± 0.53 b
Finite rate of increase (λ)	1.167 ± 0.000 b	1.187 ± 0.000 a	1.200 ± 0.000 a
Population doubling time (*DT*)	4.501 ± 0.040 a	4.052 ± 0.021 b	3.829 ± 0.032 b

Data are shown as mean ± SE. Different letters in the same row indicate significant differences (one-way ANOVA followed by Tukey’s HSD tests, *p* < 0.05).
